# Retinoblastoma in a pediatric oncology reference center in Southern Brazil

**DOI:** 10.1186/s12887-016-0579-9

**Published:** 2016-04-03

**Authors:** Simone G. A. Selistre, Marcelo K. Maestri, Patricia Santos-Silva, Lavinia Schüler-Faccini, Luis S. P. Guimarães, Juliana Giacomazzi, Mario C. Evangelista Júnior, Patricia Ashton-Prolla

**Affiliations:** Post-Graduate Program in Medicine: Medical Sciences, Universidade Federal do Rio Grande do Sul (UFRGS), Porto Alegre, Brazil; Pediatric Oncology Service, Hospital de Clinicas de Porto Alegre (HCPA), Porto Alegre, Brazil; Ophthalmology Service, HCPA, Porto Alegre, Brazil; Genomic Medicine Laboratory, Experimental Research Center, HCPA, Porto Alegre, Brazil; Post-Graduate Program in Genetics and Molecular Biology, UFRGS, Porto Alegre, Brazil; Genetics Department, Biosciences Institute, UFRGS, Porto Alegre, Brazil; Instituto de Genética Médica Populacional (INAGEMP), Porto Alegre, Brazil; Medical Genetics Service, HCPA, Porto Alegre, Brazil; Statistics and Epidemiology, UFRGS, Porto Alegre, Brazil; Statistics and Epidemiology, HCPA, Porto Alegre, Brazil; Hospital Tacchini, Bento Gonçalves, Brazil

**Keywords:** Retinoblastoma, Malignant tumors of the retina, Intraocular malignancies, Hereditary retinoblastoma, Pediatric tumors

## Abstract

**Background:**

Retinoblastoma (Rb) is the most common intraocular tumor diagnosed in children in Brazil. However, detailed information is lacking regarding patient clinical demographics. This study aimed to determine the clinical profile of patients with Rb who were treated in a public university hospital in southern Brazil from 1983 to 2012. Methods: Patients’ medical records were reviewed to retrospectively identify patients with a principal diagnosis of Rb. Rb was classified as hereditary or non-hereditary. Clinical staging was reviewed by an ophthalmologist. Statistical analysis was performed using SPSS.

**Results:**

Of 165 patients with a diagnosis of Rb during this period, 140 were included in the study. Disease was unilateral in 65.0 % of patients, bilateral in 32.9 %, and trilateral in 2.1 %. The mean age at onset of the first sign/symptom was 18.1 month, and 35.7 % of patients were diagnosed during the first year of life. The most common presenting signs were leukocoria (73.6 %) and strabismus (20.7 %). The mean age at diagnosis was 23.5 months, and time to diagnosis was 5.4 months. In patients with clinical features of hereditary Rb, both onset of the first sign/symptom and diagnosis were at an earlier age than in patients without these features (12.3 vs 21.6 months [*P* = 0.001] and 15.9 vs 28.0 months [*P* < 0.001], respectively). However, there was no significant difference in overall survival between the two groups. Ocular stage at diagnosis was advanced in 76.5 % (Reese V) and 78.1 % (International Classification D or E). Of patients with unilateral and bilateral disease, 35.2 % and 34.8 %, respectively, had extraocular disease at diagnosis; 10.7 % had metastatic disease at diagnosis. Enucleation was observed in 88.1 % and exenteration in 11.9 % of patients; 93.6 % patients were followed until 2012, and 22.9 % relapsed. Overall survival was 86.4 %.

**Conclusions:**

Most Rb diagnoses are still diagnosed in advanced stages of the disease, considerably reducing overall survival time and the rate of eye and vision preservation.

**Electronic supplementary material:**

The online version of this article (doi:10.1186/s12887-016-0579-9) contains supplementary material, which is available to authorized users.

## Background

In Brazil, cancer is the leading cause of death by disease among children and adolescents aged ≤ 19 years, with an incidence of 11,530 new cases in 2012 [1, 2]. Retinoblastoma (Rb) is the most common primary intraocular malignancy of childhood, and most cases are diagnosed before five years of age [3–5].

Rb is considered a rare tumor in developed countries, accounting for approximately 3 % of all childhood malignancies and 11 % of all tumors that develop during the first year of life [6–8]. Its global incidence is estimated at 1:12, 500–25,000 live births (1:16,000 in France) [9]. The annual incidence rate of Rb in the United Stated is 3.4 to 4.0 per million children aged 0–15 years [4, 10]. There is indirect evidence that its incidence increases in developing countries, including those in Latin America, Africa, India, and Asia (excluding Japan). Therefore, in these areas Rb is considered one of the most frequent pediatric solid tumors [8, 11].

The most common presenting sign of Rb is leukocoria (75 % of cases), followed by strabismus (25 % of cases) [12–14]. Although evidence of sex predominance is inconclusive, a few studies have shown a higher prevalence of Rb in boys (1.1–1.4:1) [4, 15]. Approximately 40 % of all Rb cases are hereditary, caused by germline mutations in the *RB1* gene [4, 7, 16]. The retinoblastoma phenotype in addition to presence or absence of family history is important features to determine the probability of hereditary predisposition. Thus, the probability of hereditary retinoblastoma in patients with bilateral, trilateral or unilateral Rb with a positive Rb family history is 90, 100 and 15 %, respectively [4, 10].

Different treatment modalities are available for patients with Rb, including cryotherapy, laser therapy, enucleation, radiotherapy, high-dose systemic chemotherapy, intra-arterial chemotherapy, intravitreal chemotherapy, and autologous stem cell transplantation. Treatment should be tailored to the patient’s needs, taking into account laterality, ocular stage, presence of extraocular disease, the child’s age, and visual acuity [17–19]. Overall, the prognosis is favorable for patients with early-stage intraocular Rb, with a 5-year survival rate of 93 %. However, when there is extraocular extension, more aggressive treatment is required and the 5-year overall survival decreases dramatically to approximately 30 % [8, 11, 18, 20–22].

Although Rb is the most common intraocular tumor in children, there are little published data regarding the general characteristics of patients diagnosed and treated in Brazil. Most data are obtained from online databases of cancer registries located mainly in the southeastern region of the country. According to the 14 registries in this region, the incidence rate of Rb, in 2010, ranged from 2.40 to 9.80 per million children and adolescents aged ≤ 19 years [23]. However, detailed information is lacking regarding patient demographics and clinical characteristics, especially in the southern region [23]. The aim of the present study was to characterize patients with a diagnosis of Rb who were treated in a public university hospital in southern Brazil, providing additional data that may contribute to improving the diagnosis and management of these patients.

## Methods

This was a retrospective cohort study of patients with Rb who were treated in the Departments of Pediatric Oncology, Ophthalmology and Medical Genetics at Hospital de Clínicas de Porto Alegre (HCPA) from 1983 to 2012. HCPA is a tertiary care teaching hospital located in Porto Alegre, city and capital of Rio Grande do Sul, the southernmost state of Brazil. The study was approved by the Institutional Review Board – Hospital de Clínicas de Porto Alegre (IRB number 100521). The need for informed consent was waived by this IRB for this retrospective and epidemiologic study.

The patients’ medical records were reviewed to retrospectively identify patients with a principal diagnosis of Rb according to the International Classification of Diseases, 10th Revision, from codes designating malignant neoplasms of the eye (codes from C69.0 to C69.9); more specifically code C69.2 (malignant neoplasms of the retina). Clinical and demographic data were also collected at this stage using a protocol developed by the authors. Rb was classified as hereditary or non-hereditary according to the clinical guidelines proposed by Lohmann and Gallie [3].

Except for patients who had undergone enucleation at another hospital before transfer, the diagnosis was confirmed by an ophthalmologist (MKM)based on data from medical records, presence of typical signs, such as leukocoria, and either (a) by binocular indirect ophthalmoscopy, with visualization of a characteristic yellow-white mass, or (b) by ocular ultrasound and computed tomography (CT) of the eye and orbit for identification of intratumoral calcification in eyes with turbid media, which prevented direct visualization of the retina. Although it is our understanding that CT-scans should be avoided in patients with retinoblastoma, for many years alternative imaging (i.e., magnetic resonance imaging) was not available in the institution and CT scans were often used. In addition, CT scans were performed in all patients for evaluation of possible metastatic disease at diagnosis. Histopathologic specimens of enucleated or exenterated eyes were further analyzed by a pathologist. Clinical staging was reviewed by same ophthalmologist (MKM) based on the Reese-Ellsworth classification and the International Classification of Intraocular Rb (ABCDE groups) [6, 22, 24]. Extraocular disease was classified according to the system used by the Children’s Cancer Group [24, 25]. Systemic staging was performed as previously described [6, 22, 26, 27]. All therapeutic procedures performed for management of Rb were recorded for each patient. The choice of initial treatment was based on protocols established by international reference centers or on the 2009 Brazilian Protocol for Rb Treatment developed by the Brazilian Society of Pediatric Oncology [26, 27]. In brief, we identified 3 different protocols of treatment used in different years along the overall study period. This information is summarized in Additional file 1: Table S1.

The following outcomes were assessed: (a) time to diagnosis, defined as the time between onset of the first sign or symptom and the actual diagnosis;(b) duration of follow-up, calculated as the difference between the patient’s age at diagnosis and their age at the time of the last consultation (if alive) or death; (c) diagnosis of a second or third neoplasm; and (d) death. Loss to follow-up was defined as no recorded consultation with a physician at HCPA for more than five years.

Statistical analysis was performed using SPSS, version 18.0, and the level of significance was set at *P* < 0.05. Continuous variables were expressed as mean (minimum-maximum), median and interquartile range (IQR), with a 95 % confidence interval. Categorical variables were expressed as absolute and relative frequencies. The Kaplan-Meier method was used to estimate survival as a function of time, and the log-rank test was used for comparison of survival curves according to clinical characteristics. The patient’s age and time to diagnosis, in association with other characteristics, were analyzed using the Kruskal-Wallis test, followed by Dunn’s multiple-comparison test when *P*-values were less than 0.05. Fisher’s exact test was used to analyze the association of disease extension (intra- or extraocular) and laterality.

## Results

### Demographics

Of 165 patients with a diagnosis of Rb who were treated at HCPA from 1983 to 2012, 140 (86.4 %) were included in the study. The medical records of25 patients who were diagnosed during the first two decades of the study were not available. Most patients (95.0 %) were born and lived in the state of Rio Grande do Sul, and 21.8 % were from the capital of the state, Porto Alegre. Tables 1 and 2 shows the main clinical characteristics of patients with Rb included in the study. There was a slight predominance of male over female patients (*n* = 87; 62.1 %).

**Table 1:** Characteristics of patients with a diagnosis of retinoblastoma (Rb) (*n* = 140)

Characteristics (months)	Mean median	95 % CI	
Age at first sign or symptom	18.1	12.0	0–129.0
Age at diagnosis	23.5	16.5	1.0–206.0
Time to diagnosis	5.4	3.0	0–77.0
Duration of follow-up	323.2		300.3–346.1

**Table 2:** Characteristics of patients with a diagnosis of retinoblastoma (Rb) (*n* = 140)

	N	%
First sign or symptom^a^		
Leukocoria	103	73.6
Strabismus	29	20.7
Glaucoma	4	2.9
Buphthalmos	4	2.9
Proptosis	4	2.9
Hyperemia	4	2.9
Ocular pain	3	2.1
Anisocoria	3	2.1
Blindness	2	1.4
Orbital edema	2	1.4
Hyphema	2	1.4
Visual deficiency	1	0.7
Cervical adenopathy	1	0.7
Ecchymosis	1	0.7
Total eyes involved	187	66.8
Ocular laterality		
Unilateral Rb	91	65.0
Right eye	50	54.9
Left eye	41	45.1
Bilateral Rb	46	32.9
Trilateral Rb	3	2.1
Systemic dissemination at diagnosis		
Non-metastatic disease	125	89.3
Metastatic disease	15	10.7
Metastatic sites at diagnosis		
Orbit	12	80.0
CNS	8	53.3
Bone	4	26.7
Bone marrow	3	20.0
Cerebrospinal fluid	2	13.3
Cervical lymph nodes	1	6.7

Legend: Ages, time to diagnosis and duration of follow-up are expressed in months; Kruskal-Wallis test was used for analysis of ages and time to diagnosis; Log-rank test was used to estimate duration of follow-up; time to diagnosis: time between onset of the first sign or symptom and diagnosis; duration of follow-up: difference between the patient’s age at diagnosis and their age at the time of the last consultation (if alive) or date of death

*CNS* central nervous system

^a^some patients had more than one sign or symptom; more than one site per patient

### Diagnosis

Most tumors were unilateral at diagnosis (*n* = 91; 65.0 %). In most cases, unilateral tumors were diagnosed at an advanced stage (*n* = 88,96.7 %; 4 at IVb stage and 84 at Va or Vb stage), and all of them were considered unifocal because of their large size. The few unilateral tumors diagnosed at an early stage (*n* = 3, IIb stage) were also unifocal. Forty-six patients (32.9 %) had bilateral lesions at diagnosis, most of which (80.4 %) were multifocal (*P* = 0.015).

There was no association between sex and disease laterality (*P* = 0.351). Similarly, there was no association between the clinical presentation of leukocoria or strabismus and poor prognosis (*P* = 0.612) or between time to diagnosis > 6 months and poor prognosis (*P* = 0.052).

Bilateral and trilateral tumors were diagnosed at an earlier age than unilateral tumors (*P* < 0.001). Fifty patients (35.7 %) were diagnosed before 12 months of age. Of these, 44.0 % had unilateral tumors and 56.0 % had bilateral tumors; 6.0 % had metastatic disease.

**Fig. 1:**
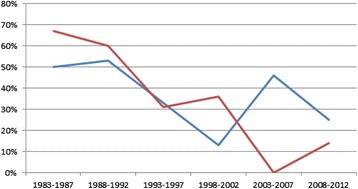
Proportion of cases of extraocular disease at diagnosis in patients with unilateral and bilateral Rb from 1983 to 2012. Legend: At the point where the two lines meet (between 1993 and 1997), 33 % refers to unilateral cases and 31 % refers to bilateral cases. *Line red: bilateral extraocular *Line blue: unilateral extraocular

Ocular staging at diagnosis is shown in Tables 3 and 4. Extraocular extension of disease in at least one eye at diagnosis was present in 32 of 91 patients (35.2 %) with unilateral Rb, in 16 of 46 patients (34.8 %) with bilateral Rb, and in all three patients with trilateral Rb, totaling 51 patients (36.4 %). Considering the total number of eyes involved (*n* = 187), 28.9 % had extraocular disease at diagnosis. Over the years, a decrease was observed in the proportion of patients with extraocular disease (Fig. 1).

**Table 3:** Ocular staging at diagnosis

Reese-Ellsworth classification						
Ocular staging	A	%	B	%	Total	(%)
I	1	0.5	3	1.6	4	2.1
II	12	6.4	8	4.3	20	10.7
III	7	3.8	3	1.6	10	5.4
IV	5	2.7	5	2.7	10	5.4
V	92	49.2	23	12.0	115	61.5
Presumed V^a^	26	13.9	2	1.1	28	15.0

^a^Patients evaluated after enucleation performed at another hospital

**Table 4:** Ocular staging at diagnosis

International Classification of Intraocular Rb (ABCDE)		
Ocular staging	N	%
A	3	1.6
B	30	16.0
C	8	4.3
D	32	17.1
E	89	47.6
Presumed D^a^	5	2.7
Presumed E^a^	20	10.7

^a^Patients evaluated after enucleation performed at another hospital

All patients were evaluated for features suggestive of hereditary Rb (Table 5). The presence of at least one criterion suggestive of hereditary Rb was observed in 52 patients (37.1 %) from 50 different families. One- and two-generation family history of Rb was positive for cancer in 23 patients (16.4 %), and 10 of these patients (43.5 %) had an affected parent. The mean age at onset of the first sign or symptom was 12.3 months in the group with probable hereditary predisposition to Rb and 21.6 months in the non-hereditary group (*P* = 0.001). The mean age at diagnosis was 15.9 months in the hereditary group and 28.0 months in the non-hereditary group (*P* < 0.001). However, there was no significant difference in overall survival between the hereditary and non-hereditary groups (84.6 % vs. 87.5 %, respectively; *P* = 0.844). Survival data are summarized in Additional file 2: Figure S1.

**Table 5:** Distribution of patients with criteria for hereditary Rb at diagnosis

Criteria for hereditary Rb	Number	Percent
Total of families with at least one criterion for hereditary Rb^a^	50	36.2
Only one criterion present:		
Bilateral^b^	39	75.0
Trilateral^c^	3	5.8
Family history of Rb^ac^	3	5.8
Two criteria present:		
Bilateral and family history^c^	7	13.4
Samples collected for mutation analysis based on criteria^d^	25	48.1
*RB1* mutation identified^e^	13	52.0
Patients with secondary malignant neoplasm^f^	2	15.4

^a^Two families with two patients with Rb; ^b^Three cases of unilateral Rb with family history of Rb (all diagnosed before 12 months of age); ^c^Total cases with family history of Rb = 10; ^d^Total patients who collected samples for molecular genetic testing = 32 (22.9 %); *RB1* mutation was detected in one patient who had no criteria for hereditary Rb at diagnosis (unilateral and unifocal); normal results (*n* = 12); no results available (*n* = 6). This percentage (48.1 %) refers to the proportion 25/52; ^e^This percentage (52.0 %) refers to the proportion 13/25; ^f^This percentage (15.4 %) refers to the proportion 2/13

Data on mean age at onset of the first signs and symptoms of Rb, mean age at diagnosis and time relapsed between onset of signs and symptoms and diagnosis are shown in Tables 6 and 7. Additional file 3: Figure S2 is a graphical representation of the overall survival data described in Tables 6 and 7.

**Table 6:** Follow-up characteristics of patients with Rb according to subgroups

Laterality (*n* = 140 patients)					
	Unilateral (*n* = 91) Age; Age dx (min-max) (months)	Bilateral (*n* = 46) Age; Age dx (min-max) (months)	Trilateral (*n* = 3) Age; Age dx (min-max) (months)		P
Age at first signs and symptoms	21.7; 15.0 (0-129.0)	10.3; 6.0 (0-84.0)	29.0; 24.0 (3.0-60.0)	[24.0]	<0.001
Age at diagnosis		13.4; 8.0 (1.0-84.0)	40.3; 26.0 (15.0-80.0)		<0.001
Time to diagnosis	28.1; 22.0 (1.0-206.0)	3.1; 2.0 (0-14.0)	11.3; 12.0 (2.0-20.0)		0.029
Duration of follow-up (months)	6.4; 3.0 (0-77.0)				
	275.6 (253.2-297.9)	334.9 (299.3-370.5)	22.3 (2.9-42.7)		<0.001
Laterality for each eye (*n* = 187 eyes involved)					
	Unilateral (*n* = 91) Age; Age dx (min-max) (months)	Bilateral (*n* = 92) Age; Age dx (min-max) (months)	Trilateral (*n* = 4) Age; Age dx (min-max) (months)		
Time to diagnosis between IO and EO in each subgroup	IO: 4.0; 2.0 (0-20.0)	IO: 2.9; 2.0 (0-14.0)	IO: 2.0; 2.0 (2.0-2.0)		
	EO: 10.8; 4.5 (1.0-77.0)	EO: 4.2; 4.0 (0-12.0)	EO: 11.3; 12.0 (2.0-20.0)		
	P= 0.003	P = 0.147	P = 0.346		
Duration of follow-up between IO and EO in each subgroup (months)	IO: 293.0 (273.3-312.8)	IO: 355.7 (334.6-376.9)	IO: 4.0 (4.0-4.0)		
	EO: 230.9 (180.4-281.4)	EO: 255.5 (174.8-336.3)	EO: 22.3 (2.9-41.7)		
	P= 0.002	P = 0.001	P = 0.317		

*dx* diagnosis, *CI* confidence interval; age, time to diagnosis and duration of follow-up are expressed in months. Kruskal-Wallis test was used for analysis of ages and time to diagnosis; Log-rank test was used to estimate duration of follow-up. Disease extension, *IO* intraocular, *EO* extraocular. Time to diagnosis: time between onset of the first sign or symptom and diagnosis (months); duration of follow-up: difference between the patient’s age at diagnosis and their age at the time of the last consultation (if alive) or date of death (months)

**Table 7:** Follow-up characteristics of patients with Rb according to subgroups

Systemic dissemination (*n*= 140 patients)			
	Metastatic (*n* = 15) Age; Age dx (min-max) (months)	Non-metastatic (*n* = 125) Age; Age dx (min-max) (months)	P
Age at first signs and symptoms	32.1; 24.0 (1.0-129.0)	16.4; 11.0 (0-84.0)	0.107
Age at diagnosis	46.5; 26.0 (2.0-206.0)	20.8; 16.0 (1.0-84.0)	0.024
Time to diagnosis	14.3; 4.0 (1.0-77.0)	4.4; 3.0 (0-26.0)	0.123
Duration of follow-up (months)	77.7 (20.6-134.7)	345.2 (325.6-364.7)	<0.001
Hereditary criteria (*n*= 140 patients)			
	Hereditary (*n* = 52) Age; Age dx (min-max) (months)	Non-hereditary (*n* = 88) Age; Age dx (min-max) (months)	P
Age at first signs and symptoms	12.3; 6.5 (0-84.0)	21.6; 14.0 (0-129.0)	0.001
Age at diagnosis	15.9; 10.5 (1.0-84.0)	28.0; 21.5 (1.0-206.0)	<0.001
Time to diagnosis	3.7; 2.0 (0-20.0)	6.5; 3.0 (0-77.0)	0.074
Duration of follow-up (months)	318.2 (280.2-356.2)	274.0 (250.8-297.2)	0.844
Disease extension for each eye (*n*= 187 eyes involved)			
	Intraocular (*n* = 133) Age; Age dx (min-max) (months)	Extraocular (*n* = 54) Age; Age dx (min-max) (months)	P
Age at first signs and symptoms	13.6; 8.0 (0-84.0)	22.7; 12.0 (0-129.0)	0.001
Age at diagnosis	16.9; 13.0 (1.0-84.0)	31.3; 24.0 (1.0-206.0)	<0.001
Time to diagnosis	3.4; 2.0 (0-20.0)	8.5; 4.0 (0-77.0)	<0.001
Duration of follow-up	352.9 (336.0-369.8)	252.7 (204.7-301.3)	<0.001

### Treatment

Several treatment modalities were used in the present cohort. Among 134 patients (95.7 %) who underwent surgery, enucleation was performed in 118 (88.1 %) and exenteration in 16 (11.9 %). Fifty-seven patients (42.5 %) were treated with enucleation alone, while 77(57.5 %) were treated with enucleation combined with some other form of treatment. There has not been a significate decline in the number of enucleations related to the different chemotherapy regimens for retinoblastoma.

Only six patients (4.3 %) did not undergo enucleation and were treated with multimodal therapy, including chemotherapy, brachytherapy, thermotherapy, and cryotherapy. Among the 77 patients treated with enucleation and some other form of therapy, 74 (96.1 %) received systemic chemotherapy, followed by orbital external beam radiotherapy alone (2.6 %) and cryotherapy alone (1.3 %). Among the 74 patients treated with enucleation and systemic chemotherapy, 48 (64.9 %) also received radiotherapy. Of all 140 patients, 80 (57.1 %) received systemic chemotherapy and 52 (37.1 %) received radiotherapy. Two patients (1.4 %) underwent autologous stem cell transplantation. The treatment modalities used in the present cohort are described in detail in Additional files 4, 5 and 6: Tables S2, S3, and S4.

Six patients (4.3 %) receiving ionizing radiation were diagnosed with a secondary malignancy: four with a soft tissue sarcoma (three of the mat sites that had been previously irradiated), one with osteosarcoma, and one with acute lymphoblastic leukemia (ALL). The patient with a diagnosis of ALL developed a third malignant neoplasm (osteosarcoma of the femur) and was the only patient treated with brachytherapy in this group. Presence of germline mutations in the *RB1* gene was investigated by whole-genome sequencing and multiplex ligation-dependent probe amplification (MLPA) in two patients with multiple solid tumors. A pathogenic mutation (p.R320X) was identified in only one of the patients. Details of these six patients that developed a secondary malignancy were: all 6 patients who had a second primary tumor treatment received systemic chemotherapy (4 with cisplatin, teneposide, vincristine, doxorubicin, cyclophosphamide, and methotrexate, cytarabine intrathecal dexamethasone and 2 with vincristine, etoposide and carboplatin. The age at diagnosis of these patients varied from 3 to 26 months and the age who received systemic chemotherapy ranged from 4 to 28 months. Five of these patients also received radiation therapy between 5 and 29 months and of those, 3 had a second primary tumor in previously irradiated area. The age of the irradiated patients who had a second primary tumor ranged between 134 and 221 months, and the patient who did not undergo radiation therapy had a second primary tumor at 24 months.

### Mortality

Overall survival was 86.4 % for the entire cohort, 92.0 % for patients with non-metastatic disease, and 40.0 % for patients with metastatic disease. Overall survival for patients with intraocular disease was 94.0 % vs 68.5 % for patients with extraocular disease. Patients with unilateral and bilateral tumors had a similar overall survival rate (approximately 88.0 %), whereas all three patients with trilateral disease died.

Among patients with unilateral Rb, overall survival was 94.9 % for those with intraocular tumors and 75.0 % for those with extraocular tumors. Among patients with bilateral Rb, overall survival was 94.5 % for those with intraocular tumors and 68.4 % for those with extraocular tumors.

### Follow up

Of 140 patients treated at the institution, 131 (93.6 %) were followed until the end of 2012 or until death; only nine patients (6.4 %) were lost to follow up. During the follow up period, 32 patients (22.9 %) relapsed (20 were non-metastatic and 12 were metastatic at diagnosis), leading to 19 deaths. Of these, 16 (84.2 %) were due to disease progression (10 were non-metastatic and nine were metastatic at diagnosis).

Mean follow up time was 323.2 months (minimum, 300.3 months; maximum, 346.1 months). The mean age at diagnosis of patients with trilateral disease was 40.3 months (median, 26.0 months; IQR, 15.0–80.0 months), and mean follow up time was 22.3 months (IQR, 2.9–41.7 months).

## Discussion

The main purpose of the present study was to determine the clinical profile of patients diagnosed with Rb and treated in a public university hospital in southern Brazil over a 30-year period. Most patients were from Rio Grande do Sul, the southernmost state of Brazil. Consistent with previous findings, there was a slight predominance of male children in the population studied. However, there was no significant difference between sexes regarding laterality of the affected eye. The most common presenting signs and symptoms were leukocoria and strabismus, which are consistent with the literature [12–14]. However, there was no significant association of patients with strabismus or leukocoria at diagnosis with time to diagnosis > 6 months, and no association of any of these factors with poor prognosis as previously reported [18, 28, 29]. Since this is a retrospective study, we were unable to obtain detailed information on the exact dates of medical consultations before the diagnosis, although we expect to exist significant heterogeneity in the individual times to seek medical care.

Although most patients had unilateral disease at diagnosis, almost all patients were diagnosed at a very advanced stage (stage IVb, Va, or Vb). Early diagnosis is directly related to how easily patients are able to get needed care through the public health system and how readily health care professionals can recognize the signs and symptoms of Rb. Despite a growing economy, in Brazil, the number of patients presenting with locally advanced or metastatic disease is similar to the numbers reported for lower-income countries, with an average per capita income of USD 935.00 [30]. Conversely, about one-third of patients had bilateral disease at diagnosis, and most of these lesions were multifocal and diagnosed at an earlier age, as expected is cases of hereditary disease. However, although prevalent, the cases of hereditary Rb were not referred for genetic risk assessment. The average age at diagnosis of patients with hereditary Rb according to our clinical criteria (12.3 months) was similar to the average age of diagnosis in studies from developed countries (12 months) [6]. Only a proportion of the patients with probable hereditary Rb were offered genetic tests to evaluate *RB1* mutations treatment options. Only a few patients with probable hereditary Rb were offered genetic testing for *RB1* mutations, and several patients were treated with radiotherapy because of the lack of other treatment options. This may be due to difficulties in accessing public health services, but at least in part it also reflects a lack of knowledge about how hereditary Rb manifests itself. It is important to note that molecular genetic testing is not available to patients receiving care through the public health system in Brazil. Such tests are performed only at the Brazilian National Cancer Institute for patients who are currently enrolled in clinical trials or research protocols [1].

Based on tumor characteristics, ocular stage was advanced in a large number of patients (stage V in 76.5 %; D or E in 78.1 %) and metastatic disease was present at diagnosis in 10.7 % of cases. These figures are much higher than those expected for developing countries with a socioeconomic status equivalent to that of Brazil [8, 31, 32]. Possible explanations for this result include inability of health care professionals to recognize the signs of Rb and difficult access to specialized pediatric oncology centers for Rb diagnosis and treatment [8, 30–33]. In another Brazilian study, conducted in São Paulo, 83 pediatric patients with a diagnosis of extraocular Rb admitted for treatment between 1987 and 2000 were treated with two different chemotherapeutic regimens. The authors reported an average age at diagnosis of 32.9 months, with unilateral disease presented with a median age of 33.2 months and those with bilateral disease had a median age of 23.7 months and a latency of 10.5 months between the appearance of the first sign and confirmation of diagnosis [33]. Comparatively, in the present study, the mean age at diagnosis was 23.5 months, but with a time of 34.1 months which may be due to the unilateral cases (unilateral 41.3 vs. bilateral 10.6 vs. trilateral 11.3 months) [33]. A delayed diagnosis can be expected in atypical cases where these usual signs of presentation, leukocoria, strabismus and posterior pole tumors are not present.

Only 16.4 % of patients in the present cohort had a positive family history of cancer, which is below the estimated rate of 30 % [1, 16, 34]. However, 37.1 % of patients had at least one criterion for hereditary Rb, which is consistent with the rate of 40 % described in the literature [4, 7, 10, 16]. As expected, patients with criteria for hereditary Rb showed typical signs and were diagnosed at an earlier age, the germline mutation in the *RB1* gene [34]. Thus, early detection could also be attributed in part to a closer observation by parents who were aware of the family history. There was no difference in overall survival between the hereditary and non-hereditary subgroups, which may be due to prompt and appropriate intervention.

Late diagnosis at an advanced stage, which was predominant in the present cohort was probably the main determining factor in the choice of enucleation as the primary treatment, with a large proportion of cases indicated for surgical procedure: approximately 60 % of the eyes were enucleated, and 12 % exenterated. Even considering modern globe salvage treatment options in retinoblastoma, enucleations is still high in unilateral cases, because the majority was diagnosed in advanced D and E stages. It is estimated that 30 % of all Rb cases worldwide occur in sub-Saharan Africa, where the population faces considerable barriers to access health care. Two African studies, conducted over the past 5 years, reported enucleation rates of 67 and 64 %, with overall 30-month survival rates of 56 and 36 %, respectively [9, 35]. Several lines of evidence show a clear correlation between a country’s socioeconomic status and the incidence of extraocular disease, metastatic disease, and overall survival. In this sense, the present findings regarding disease stage at diagnosis and overall survival are comparable to those for patients from low-income countries. Although a slight decrease in the frequency of extraocular disease at diagnosis was observed over time, the current scenario is still far from what is observed in developed countries (5-year overall survival of 95 %, and metastatic disease present in less than 10 % of patients) [31, 36]. As an illustration, the chart below comparatively shows the rates of intraocular disease, extraocular disease, and metastatic disease in three countries with different socioeconomic profiles [36] and the results obtained in the present. Tanzania, India and Argentina are considered countries with a low, average and high socioeconomic status, respectively, according to the World Bank [36].

The most important prognostic variable observed in this study is late diagnosis and the advanced tumor staging is the main determinant for choosing which treatment will be employed. Enucleation in D unilateral disease classified patient is a reasonable treatment option to avoid prolonged systemic chemotherapy and its relevant potential sequelae we still observe an elevated percentage of cases treated with radiotherapy (37.1 %), even in patients with a clinical presentation suggestive of hereditary Rb, for whom the treatment of choice should be systemic chemotherapy [31]. Radiotherapy is an option of conservative treatment for retinoblastoma vitreous seeding, mainly in bilateral disease, but this clinical sign is related to bad prognosis regarding globe preservation. So, in such situation, enucleation is an ultimate safe treatment option and must not be considered as a failure.

An additional barrier is that only a few centers in Brazil have access to other therapeutic procedures, such as brachytherapy and intra-arterial chemotherapy, which are indicated as a treatment for early-stage tumors and allow preservation of the globe [7, 31, 37]. Moreover, in the present cohort, only two patients underwent autologous stem cell transplantation, although several other patients were good candidates for this procedure [18, 38–41].

According to the characteristics observed at diagnosis, the overall survival rate (86.4 %) and survival rate within the non-metastatic (92 %) and metastatic (40 %) subgroups were similar to those reported in the literature [1, 5, 18, 31]. No clear, systematic treatment was offered during the first 15 years of the study. However, this behavior changed with the implementation of treatment protocols established by Brazilian cooperative groups in the last decade [26, 32]. This initiative is in accordance with the guidelines of the International Society of Pediatric Oncology, which published in 2013 the recommendations for staging and treatment of unilateral and bilateral Rb in developing countries [32]. Other similar initiatives have been implemented in specific regions, including Latin America, aiming to reduce mortality by education, early diagnosis, and proper treatment of Rb [18, 19, 28, 42].

## Conclusion

Considering that Rb is the most common intraocular neoplasm in children in Brazil and that most Rb diagnoses are still made at advanced stages of the disease, resulting in a considerable reduction in overall survival time and in the rate of eye and vision preservation, several initiatives are needed to change this scenario. These include raising public awareness and training of health care professionals for early recognition of the signs and symptoms of Rb, improving access to health care, such as prompt consultation with an ophthalmologist and a pediatric oncologist, and facilitating access to pediatric oncology centers, where specialized treatment can be provided. Even in the absence of suspicious signs or symptoms, parents should take their children to an ophthalmologist every three months during their first year of life, as recommended by the Brazilian Society of Ophthalmology and the Brazilian Society of Pediatrics.

Early diagnosis of Rb is the main goal and challenge for health care professionals in order to offer these patients a chance to achieve cure, similar to what is currently observed in developed countries.

## Additional files

Additional file 1: Table S1. Chemotherapy protocols used during the period of study. (DOCX 14 kb) Additional file 2: Figure S1. Overall survival of patients with presumed hereditary and non-hereditary Rb (TIFF 36 kb) Additional file 3: Figure S2. Overall survival (2A, 2B, 2C) of patients with Rb divided into groups according to laterality, disease extension, and systemic dissemination. (TIFF 62 kb) Additional file 4: Table S2. Surgical treatments performed in patients with retinoblastoma (Rb). (*N* = 140 patients). (DOC 29 kb) Additional file 5: Table S3. Local treatments performed in patients diagnosed with retinoblastoma (Rb) - (*N* = 140 patients). (DOCX 15 kb) Additional file 6: Table S4. Systemic treatments performed in patients with retinoblastoma (Rb). (*N* = 140 patients). (DOCX 14 kb)

## Abbreviations

ALL: Acute lymphoblastic leukemia
CT: computed tomography
HCPA: Hospital de Clínicas de Porto Alegre
IQR: interquartile range
MLPA: multiplex ligation-dependent probe amplification
Rb: retinoblastoma
SPSS: statistical package for the social sciences
