# Supplementary material for: Retinoblastoma in a pediatric oncology reference center in Southern Brazil
Source: BMC Pediatr. 2016 Apr 3;16:48. doi: 10.1186/s12887-016-0579-9 (PMC4818960; doi:10.1186/s12887-016-0579-9)
Supplement: Additional file 1: Table S1. — Chemotherapy protocols used during the period of study. (DOCX 14 kb) [file 12887_2016_579_MOESM1_ESM.docx]

**Table S1. Chemotherapy protocols used during the period of study.**

| Period of time | Patients included (N) | Chemotherapy Protocol |
| --- | --- | --- |
| 1983-1996 | 65 | CiT/VDC/MADit^1^ |
| 1997-2004 | 46 | VEC^2^ |
| 2005-2012 | 29 | VEC and VC^3^ |

Legend: 1. Cisplatin, Teniposide / Vincristine, Doxorubicin,

Cyclophosphamide / MTX, Cytarabine and Intrathecal Dexamethasone;

2. Vincristine,Etoposide, Carboplatin; 3. Vincristine, Carboplatin.
