# Supplementary figures and images for: Retinoblastoma in a pediatric oncology reference center in Southern Brazil
Source: BMC Pediatr. 2016 Apr 3;16:48. doi: 10.1186/s12887-016-0579-9 (PMC4818960; doi:10.1186/s12887-016-0579-9)

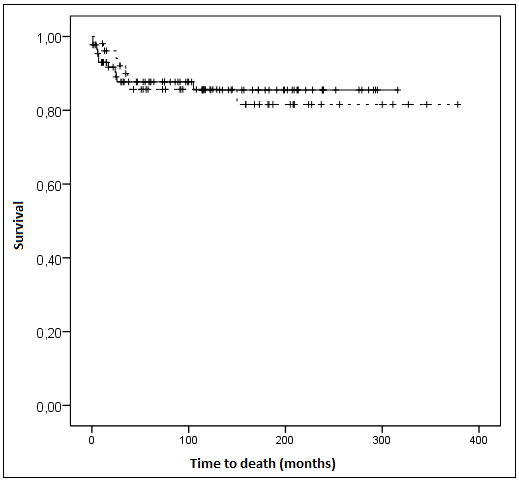

Supplement: Additional file 2: Figure S1. — Overall survival of patients with presumed hereditary and non-hereditary Rb (TIFF 36 kb) [file 12887_2016_579_MOESM2_ESM.tiff]

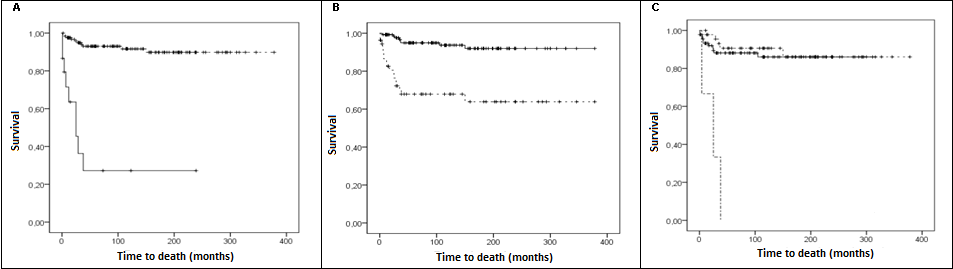

Supplement: Additional file 3: Figure S2. — Overall survival (2A, 2B, 2C) of patients with Rb divided into groups according to laterality, disease extension, and systemic dissemination. (TIFF 62 kb) [file 12887_2016_579_MOESM3_ESM.tiff]
