# Supplementary material for: Retinoblastoma in a pediatric oncology reference center in Southern Brazil
Source: BMC Pediatr. 2016 Apr 3;16:48. doi: 10.1186/s12887-016-0579-9 (PMC4818960; doi:10.1186/s12887-016-0579-9)
Supplement: Additional file 4: Table S2. — Surgical treatments performed in patients with retinoblastoma (Rb). (N = 140 patients). (DOC 29 kb) [file 12887_2016_579_MOESM4_ESM.doc]

**Supplementary table 2. Surgical treatments performed in patients with retinoblastoma (Rb). (N = 140 patients).**

| Treatments | N | % |
| --- | --- | --- |
| Surgery¹  Enucleation  Exenteration  Surgery in one eye  Surgery in second eye  As first treatment  After relapse of other eye  Surgery alone  Treatments combined with surgery  Cryotherapy  Chemotherapy  Chemotherapy and external radiotherapy  External radiotherapy | 134  118  16  120  14  7  7  57  1  74  48  2 | 95.7  88.1  11.9  89.6  10.4  42.5  0.7  55.2  35.8  1.5 |

Legend: Of all 140 patients, 6 did not undergo surgery.
