# Supplementary material for: Retinoblastoma in a pediatric oncology reference center in Southern Brazil
Source: BMC Pediatr. 2016 Apr 3;16:48. doi: 10.1186/s12887-016-0579-9 (PMC4818960; doi:10.1186/s12887-016-0579-9)
Supplement: Additional file 5: Table S3. — Local treatments performed in patients diagnosed with retinoblastoma (Rb) - (N = 140 patients). (DOCX 15 kb) [file 12887_2016_579_MOESM5_ESM.docx]

**Supplementary table 3. Local treatments performed in patients diagnosed with retinoblastoma (Rb) - (N = 140 patients).**

| Treatments | N | % |
| --- | --- | --- |
| Cryotherapy^1^  Cryotherapy and surgery  Cryotherapy and thermotherapy    Thermotherapy^2^  Thermotherapy and surgery  Thermotherapy and cryotherapy    External radiotherapy of the orbit^3^  Radiotherapy and surgery    Brachytherapy  Local relapse which required external radiotherapy of the orbit  afterwards  Radiotherapy of metastatic lesions  CNS^4^  CNS and neuro axis | 15  12  5  10  7  5  52  50  4  2  4  2  2 | 10.7  7.1  37.1  2.9  2.9 |

Legend: 1. in one eye - N = 10; in both eyes - N = 5; 2. in one eye - N = 5; in both eyes - N = 5; 3. in one eye N = 49; in boths eyes N = 3; 4. CNS = Central nervous system.
