# Supplementary material for: Retinoblastoma in a pediatric oncology reference center in Southern Brazil
Source: BMC Pediatr. 2016 Apr 3;16:48. doi: 10.1186/s12887-016-0579-9 (PMC4818960; doi:10.1186/s12887-016-0579-9)
Supplement: Additional file 6: Table S4. — Systemic treatments performed in patients with retinoblastoma (Rb). (N = 140 patients). (DOCX 14 kb) [file 12887_2016_579_MOESM6_ESM.docx]

**Supplementary table 4. Systemic treatments performed in patients with retinoblastoma (Rb). (N = 140 patients).**

| Treatments | N | % |
| --- | --- | --- |
| Systemic Chemotherapy  Chemotherapy, and surgery  Chemotherapy, surgery and external radiotherapy  Chemotherapy, surgery, external radiotherapy,  cryotherapy and thermotherapy  Chemotherapy, surgery, cryotherapy, and  thermotherapy  Chemotherapy, surgery and cryotherapy  Chemotherapy, surgery and thermotherapy  Bone Marrow Transplant  Indication:  Trilateral Rb  Extraocular unilateral Rb with metastases in cervical  lymphonode | 80  74  48  2  3  2  1  2  1  1 | 57.1  92.5  60.0  2.5  3.7  2.5  1.2  1.4 |
